# Size-, electric-field-, and frequency-dependent third-order nonlinear optical properties of hydrogenated silicon nanoclusters

**DOI:** 10.1038/srep28067

**Published:** 2016-06-16

**Authors:** Haipeng Li, Hu Xu, Xiaopeng Shen, Kui Han, Zetong Bi, Runfeng Xu

**Affiliations:** 1 Department of Physics, China University of Mining and Technology, Xuzhou, 221116, China; 2 Department of Physics, South University of Science and Technology of China, Shenzhen, 518055, China

## Abstract

We investigated the electronic properties and second hyperpolarizabilities of hydrogenated silicon nanoclusters (H-SiNCs) by using the density functional theory method. The effects of cluster size, external electric field and incident frequency on the second hyperpolarizability were also examined, respectively. We found that small H-SiNCs exhibit large second hyperpolarizability. With the increase of the number of silicon atoms in H-SiNCs, the frontier molecular orbital energy gap decreases, attributed to the enhancement of the second hyperpolarizability. Interestingly, we also found the electric-field-induced gigantic enhancement of the second hyperpolarizability for H-SiNCs due to the change of electron density distributions. In addition, our results demonstrate a significant dependence on the frequency of incident light.

Nonlinear-optical (NLO) properties in silicon-based materials have been a greatly attractive subject of many experimental and theoretical studies in recent years since they can be potentially applied in optoelectronic and photonic devices by using mature silicon technology^1^,^2^,^3^. Bulk crystalline silicon has a weak NLO effect that restricts its actual applications^4^. In the nanometer scale, however, silicon shows significantly different optical properties from its bulk state because of quantum confinement^5^. Recently, the enhanced NLO effect in the near-infrared spectral range has been observed in nanocrystalline silicon films, and all-optical switching as well as optical amplifiers based on silicon nanocrystals have been realized^4^,^6^,^7^. For example, Prakash *et al*. reported a size-dependent NLO response in nanocrystalline silicon films, and they attributed it to the increase of oscillator strengths due to the localization of electron-hole pairs induced by quantum-confinement effect^6^. Also, the localized defect states are proposed to affect the NLO properties^4^. For future applications, such as all-optical regeneration, all-optical switching, and wavelength conversion, it would be desirable to fabricate silicon-based optoelectronic devices directly using silicon nanocrystals to meet the key requirement of massive manufacturability.

In addition, silicon nanoclusters (SiNCs) have also attracted much attention recently^8^. Because the study of hydrogenated SiNCs (H-SiNCs) is essential for understanding the optical properties of silicon nanocrystals, bulk-like hydrogen saturation Si_*n*_H_*m*_ clusters have been the subject of experimental^9^ and theoretical^10^ research. Although many studies reported the quantum-confinement effect on the linear optical properties of H-SiNCs^11^,^12^,^13^, NLO properties have been shown to be more complicated and less systematically studied^14^. Furthermore, it is documented that the NLO properties of materials are strongly dependent on the microstructures as well as the measurement conditions, including the external (pump) electric field^15^,^16^,^17^ and the frequency of incident laser light^18^,^19^. In particular, Nakano and co-workers reported that application of a static electric field results in a gigantic enhancement of the third-order NLO response in symmetric diradicals^17^. Little is known, however, about the size, electric field, and frequency dispersion effects on the third-order NLO properties of the SiNC-based materials. Very recently, Xu *et al*.^20^ theoretically studied the energy and structures of 10 interesting H-SiNCs with perfect *sp*^3^ hybridization: Si_10_H_16_, Si_14_H_20_, Si_18_H_24_, Si_22_H_28_, Si_26_H_30_, Si_30_H_34_, Si_35_H_36_, Si_39_H_40_, Si_44_H_42_, and Si_48_H_46_. They examined the existence of these stable clusters with magic numbers at the local minima of the formation energies. However, the electronic and third-order NLO properties of these H-SiNCs have been unclear so far. Therefore these stable H-SiNCs are good models for investigating the NLO properties of SiNC-based materials.

In the present work, we systematically investigate the third-order NLO properties of H-SiNCs (see Fig. 1) based on the first-principles method. We aim to discover the relationship between the cluster size and the second hyperpolarizability for the H-SiNCs studied, and also to assess the effects of external electric field and incident frequency on the second hyperpolarizability. Our theoretical results reveal that the third-order NLO properties of H-SiNCs are strongly dependent on the size, external electric field, and incident frequency, respectively. Our study may propose new strategies for enhancing the third-order NLO response of silicon-based nanomaterials by modulating the external electric field and the frequency of incident light.

## Results

Generally, the energy values of the highest occupied molecular orbital (HOMO), the lowest unoccupied molecular orbital (LUMO), and their energy gap *E*_*HL*_ reflect the chemical activity of the cluster, which play an important role in the electronic and optical properties^21^. Figure 2 shows the energy distribution around the HOMO and LUMO under the external electric field in the *x* direction (i.e., *F*_*x*_) for Si_10_H_16_. We found that the applied electric field induces the decrease of the gap *E*_*HL*_ as a result of the increase (decrease) of the HOMO (LUMO) energy, and also leads to enhancement of the second hyperpolarizability. For the Si_10_H_16_ cluster, the energy gap *E*_*HL*_ and the average second hyperpolarizability <*γ*> are found to be 6.88 eV and 46.58 × 10^−61^ C^4^m^4^J^−3^, respectively. Moving from the zero-field case to *F*_*x*_ = 0.01 and 0.03 au, the *E*_*HL*_ value reduces by ~92% and ~67%, whereas the <*γ*> value increases by ~116% and ~1140%, respectively. Similar results were also found in the case of *F*_*y*_ and *F*_*z*_ due to the high symmetry in the Si_10_H_16_ cluster (see Supplementary Table S1). The interesting electric-field effect mainly originates from changes in electron-density distribution induced by the external electric field^15^. In Fig. 2, we also found that the electric field strongly affects the frontier molecular orbitals. When an electric field is applied, the lobes in the positive direction of the electric field are reduced, and the lobes in the negative direction of the field are enlarged in the HOMO. For strong *F*_*x*_ = 0.03 au, the lobes of the LUMO are powerfully pushed along the positive direction of the electric field. This may be beneficial to enhancement of the charge transfer along the direction of electric field, as shown in Fig. 3. In Fig. 3, the cyan and purple-gray parts represent the regions where electron density is increased and decreased after including the external electric field *F*_*x*_, respectively. It can be seen that although the cyan and purple-gray parts interlace with each other in the *x* direction, the overall trend is that charge is transferred to the negative side of the *x* axis (namely, toward the source of the electric field). Interestingly, electron density distributions are more highly polarized when a stronger electric field is applied, which contributes to the higher NLO response.

Figure 4 shows the energy gap *E*_*HL*_ for differently sized H-SiNCs studied. With the increase of the number of silicon atoms in the H-SiNCs studied, the HOMO energy increases, whereas the LUMO energy decreases, resulting in the energy gap *E*_*HL*_ being reduced and approaching the bulk Si direct bandgap of 3.4 eV at the gamma point^22^. The gap values are approximately inversely proportional to the cluster size, in agreement with the earlier report in ref. 23. Therefore, it is possible to tailor the energy gap in such clusters by rational variation of cluster size. Our previous study revealed that the gap *E*_*HL*_ is a critical parameter in determining molecular NLO properties, and an electronic system with a larger *E*_*HL*_ should be less NLO active than with a smaller gap^24^. The size effect of the second hyperpolarizability for the H-SiNCs studied is given in Fig. 5. It is shown that the average second hyperpolarizability <*γ*> increases with the increase of the number (*N*) of silicon atoms due to the decrease of energy gap *E*_*HL*_. Interestingly, a linear relationship between <*γ*> and *N* is also found for our studied clusters. The revealed linear relationship extends our understanding of the relationship of structures and third-order NLO response for small SiNCs. Usually, the macroscopic, static third-order susceptibility *χ*^(3)^ can be described by the following relation, where local-field corrections in the Lorentz approximation are taken into account:^25^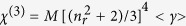, where *M* is the number density of clusters, *n*_*r*_ is the refractive index of the cluster, and <*γ*> is the average static second hyperpolarizability. Based on this approximation, we estimated the static third-order susceptibilities *χ*^(3)^ for the H-SiNCs studied to be on the order of 10^−11^–10^−12 ^esu, which is in good agreement with the experimental value of 0.5 × 10^−12 ^esu for silicon nanocrystals^26^.

We also examined the incident-frequency (ω) effect on the second hyperpolarizability. Figure 6 shows the dynamic second hyperpolarizability of Si_10_H_16_ with 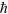
ω varying from 0.0 to 3.2 eV. We found that with the increase of ω, <*γ*(−ω; ω, 0, 0)> [corresponding to the electro-optical Kerr effect (EOKE)] and <*γ*(−2ω; ω, ω, 0)> [corresponding to electric-field-induced second-harmonic generation (EFISHG)] disperse and increase to a different extent, respectively. In particular, moving from 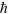
ω = 0.0 to 3.2 eV, the value of <*γ*> increases by ~7.11 times for EFISHG and ~1.67 times for EOKE. According to the sum-over-states expression for the second hyperpolarizability, <*γ*(−ω; ω, 0, 0)> (<*γ*(−2ω; ω, ω, 0)>) begins to disperse and exhibits a large value owing to the one-photon (two-photon) resonance that occurs when 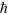
ω (2
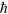
ω) is close to the strong allowed transition energy^27^,^28^. For the Si_10_H_16_ cluster, the calculated first strong allowed transition energy is about 6.53 eV. Therefore, at 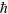
ω = 3.2 eV, the near two-photon resonance results in a larger dispersion in the <*γ*(−2ω; ω, ω, 0)> values. Our results show that the third-order NLO properties of SiNCs are strongly affected by the frequency of incident light, and thus can be tuned using the incident frequency for applications.

## Conclusions

In summary, this work revealed the effects of size, external electric field and frequency dispersion on the electronic properties and the second hyperpolarizabilities of H-SiNCs by using first-principles methods. It was found that with the increase of the number of silicon atoms in H-SiNCs, the frontier molecular orbital energy gap decreases, attributed to the enhancement of the second hyperpolarizability. In particular, a linear relationship between the second hyperpolarizability and the number of silicon atoms is found for the clusters studied. In addition, we also found the external electric field has a great influence on the frontier molecular orbital energy gap, electron-density distributions, and second hyperpolarizability. It is interesting to note that an external electric field can remarkably enhance the third-order NLO response due mainly to the fact that the strong electric-field-induced redistribution of the electron density can largely reduce the frontier molecular orbital energy gap. Finally, our results show the H-SiNCs studied exhibit significant dependence on the frequency of incident light. Therefore, our results demonstrate that the external electric field and incident frequency are good strategies for enhancing NLO response in SiNC-based materials. This study is expected to provide valuable information with which to better understand the NLO properties of silicon nanocrystals, and thus aid in designing optoelectronic devices based on them.

## Methods

**Figure 1:**
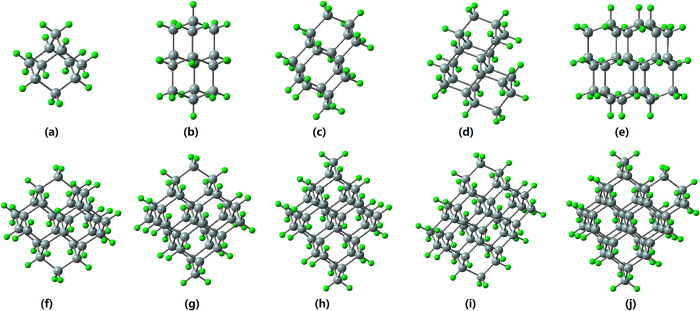
Optimized structures of H-SiNCs studied. (**a**–**j**): Si_10_H_16_, Si_14_H_20_, Si_18_H_24_, Si_22_H_28_, Si_26_H_30_, Si_30_H_34_, Si_35_H_36_, Si_39_H_40_, Si_44_H_42_, and Si_48_H_46_, respectively.

**Figure 2:**
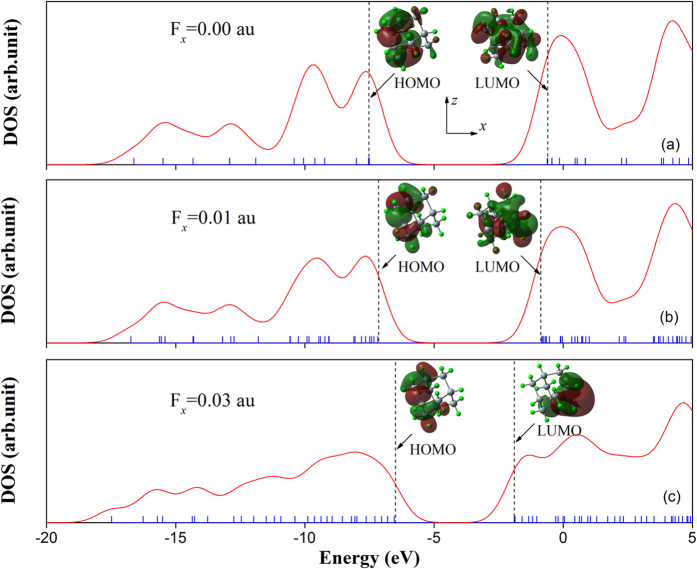
Energy density of state (DOS) and molecular orbitals (MOs) of Si_10_H_16_ under external electric fields of (**a**) *F*_*x*_ = 0.00 au, (**b**) *F*_*x*_ = 0.01 au, and (**c**) *F*_*x*_ = 0.03 au. For electric fields, 1 au = 50 V/Å. In the DOS maps, each discrete vertical blue line near the abscissa axis corresponds to a MO, and the vertical black dashed lines highlight the positions of the HOMO and LUMO, respectively.

**Figure 3:**
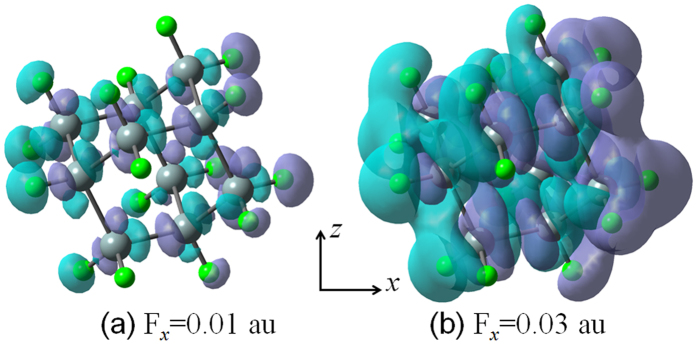
Plots of the electric-field-induced charge-density variations with respect to the zero-field case for Si_10_H_16_, (**a**) *F*_*x*_ = 0.01 au, and (**b**) F_*x*_ = 0.03 au. Purple-gray surfaces indicate regions that have gained charge with respect to the zero-field case, while the cyan surfaces indicate charge depletion. Plotted surfaces correspond to the isovalue of 0.001 e/Bohr^3^.

**Figure 4:**
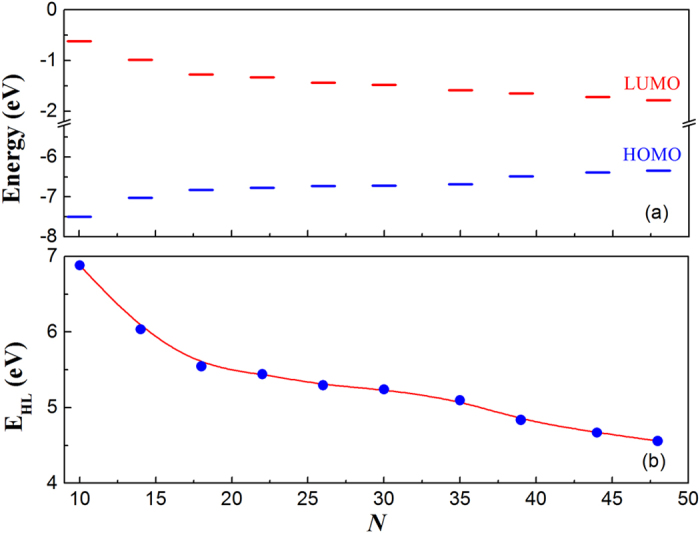
(**a**) Energies of HOMO and LUMO and (**b**) energy gap *E*_*HL*_ as a function of the number (*N*) of silicon atoms for H-SiNCs.

**Figure 5:**
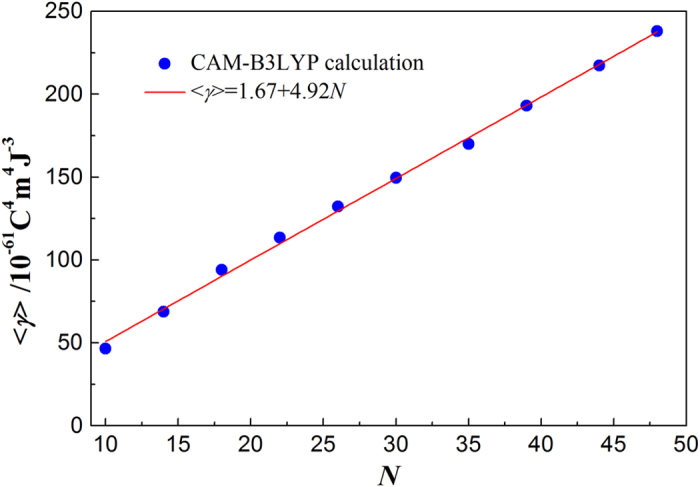
Average second hyperpolarizability <*γ*> as a function of the number (*N*) of silicon atoms for H-SiNCs.

**Figure 6:**
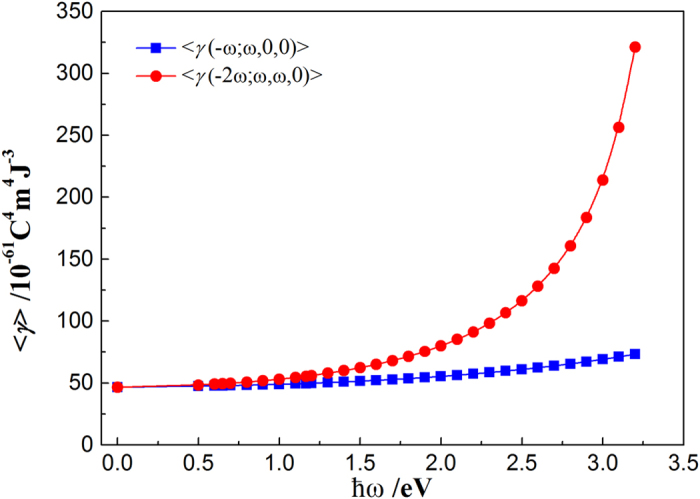
Frequency dependencies on <*γ*> of Si_10_H_16_ for EOKE and EFISHG, respectively.

We used 10 stable diamond, bulklike hydrogen saturation Si_*n*_H_*m*_ clusters^20^ as models. The optimized ground-state geometrical structures of these H-SiNCs were obtained by using the density functional theory (DFT) with the B3LYP functional^29^ and the 6–31 G(d) basis set, which are shown in Fig. 1. Then, the HOMO and LUMO energies are also computed at the B3LYP/6–31G(d) level of theory. The optimized geometries show that the Si–Si bond length is not sensitive to cluster size while it fluctuates within a small range around 2.35–2.37 Å for the H-SiNCs studied, which is close to the bulk Si–Si bond length of 2.352 Å^30^. First-principles techniques coupled with the finite-field (FF) approach are broadly applied to the investigation of NLO response because it can be used in concert with various electronic-structure methods to compute NLO coefficients^31^. In the FF method, when a system is in a weak and static electric field, its energy can be written as


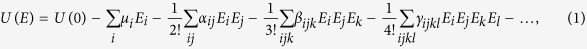


where *U*(0) is the total energy without the electric field, *E*_*i*_ is an electric-field component along the *i*th direction, *μ*_*i*_ is the component of the dipole moment vector, *α* is the linear polarizability tensor, and *β* and *γ* are the first and second hyperpolarizability tensors, which are the origins of the macroscopic second- and third-order NLO susceptibilities. Microscopically, *β* is a third-rank tensor that contains nonvanishing elements only for a noncentrosymmetric structure, while the third-order NLO effects are described by the *γ* tensor. The average second hyperpolarizability is defined as follows


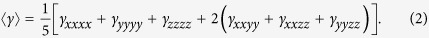


Regarding the calculation of the hyperpolarizabilities, selection of a suitable method is very important. DFT is an excellent tool to calculate the NLO properties of a large-sized system at low computational cost while including electron-correlation effects^32^. Basis-set effects and functional dependence are important for the DFT calculations of hyperpolarizability, which have been discussed in our previous work^33^. For clarity, here we only take the CAM-B3LYP functional^34^ with the 6–31 + G(d,p) basis set as an example to shed light on the effects of size, external electric field and frequency dispersion on the electronic properties and second hyperpolarizabilities of the studied clusters, respectively. All of the calculations in this work were carried out by using the Gaussian 09 program package^35^.

## Additional Information

**How to cite this article**: Li, H. *et al*. Size-, electric-field-, and frequency-dependent third-order nonlinear optical properties of hydrogenated silicon nanoclusters. *Sci. Rep.* **6**, 28067; doi: 10.1038/srep28067 (2016).

## Supplementary Material

Supplementary Information
