# Supplementary material for: Size-, electric-field-, and frequency-dependent third-order nonlinear optical properties of hydrogenated silicon nanoclusters
Source: Sci Rep. 2016 Jun 16;6:28067. doi: 10.1038/srep28067 (PMC4910094; doi:10.1038/srep28067)
Supplement: Supplementary Information [file srep28067-s1.pdf]

Size-, electric-field-, and frequency-dependent third-order nonlinear optical properties of hydrogenated silicon nanoclusters

Haipeng Li[1], Hu Xu[2], Xiaopeng Shen[1], Kui Han[1], Zetong Bi[1] & Ruifeng Xu[1]

[1]Department of Physics, China University of Mining and Technology, Xuzhou, 221116, China

[2]Department of Physics, South University of Science and Technology of China, Shenzhen, 518055, China

Table S1: Effects of electric field F_i on the energy gap E_{HL} and the average second hyperpolarizability $<\gamma>$ for the $Si_{10}H_{16}$ cluster

F_i /au	E_{HL} /eV	$<\gamma>$ /10^{-61} $C^4m^4J^{-3}$
F_x=0.00	6.88	46.58
F_x=0.01	6.35	54.24
F_x=0.03	4.64	530.80
F_y=0.00	6.88	46.58
F_y=0.01	6.34	54.18
F_y=0.03	4.47	694.25
F_z=0.00	6.88	46.58
F_z=0.01	6.35	54.25
F_z=0.03	4.62	539.57
